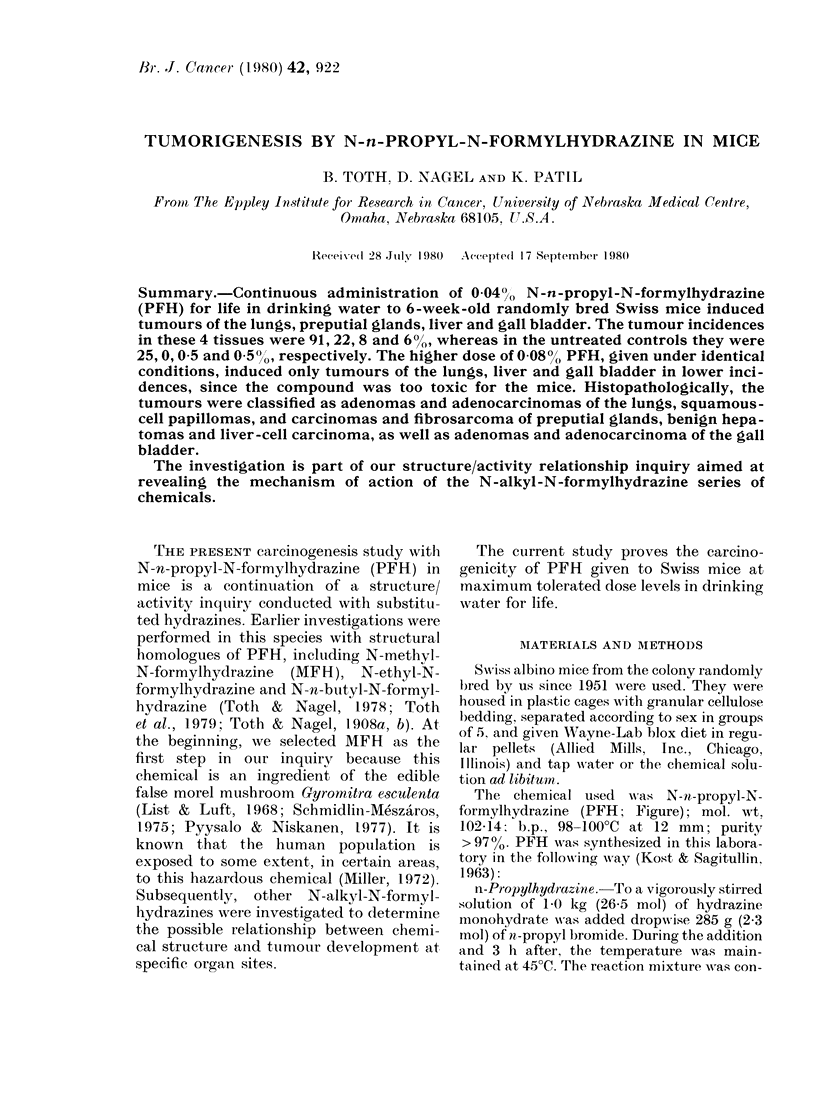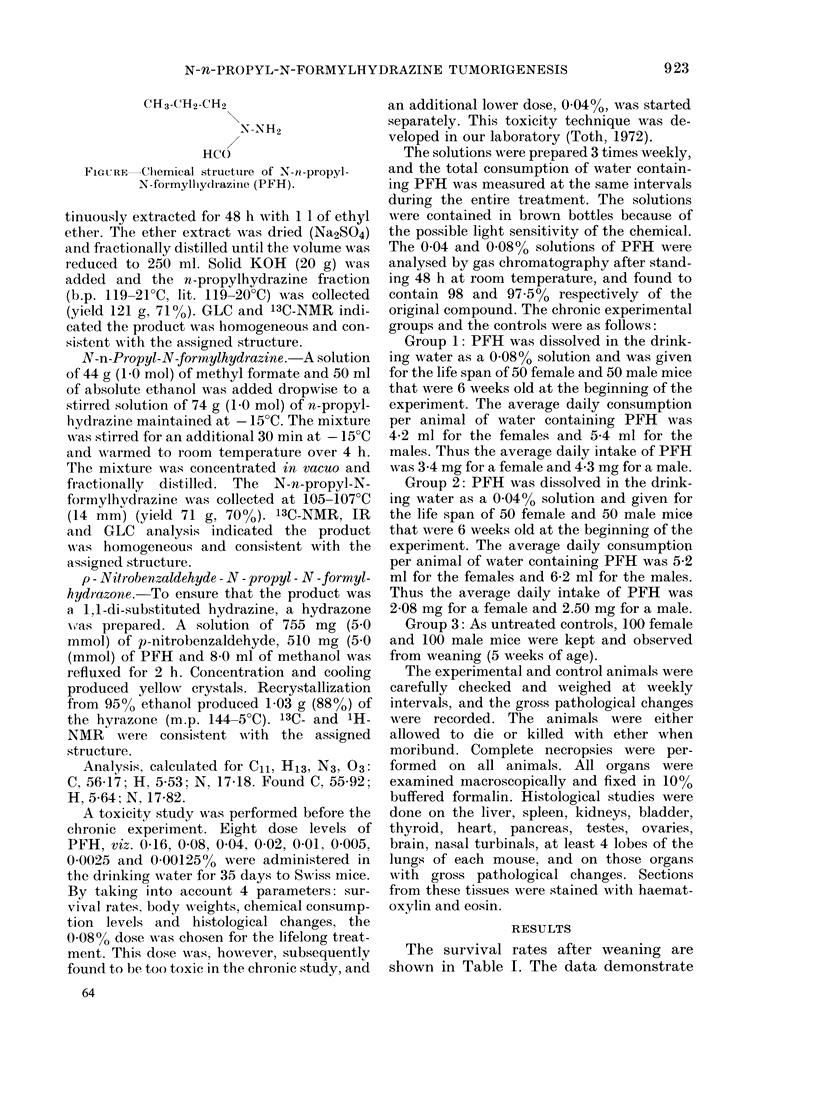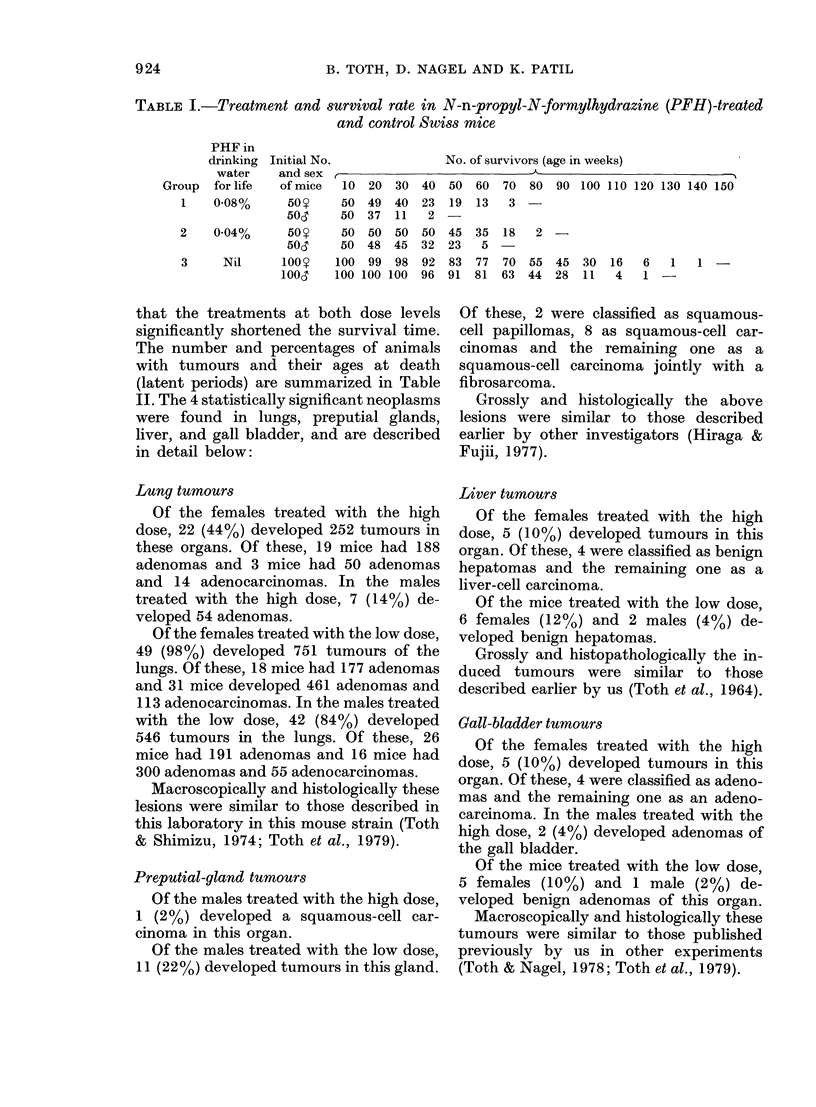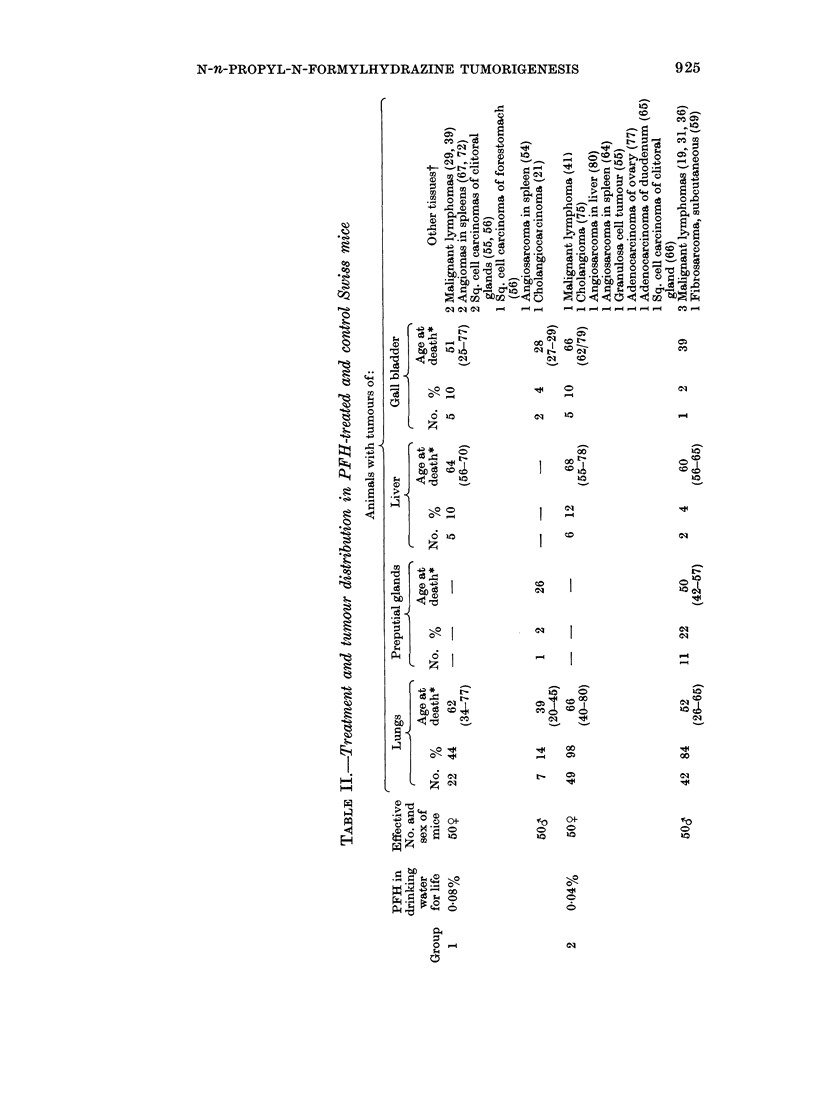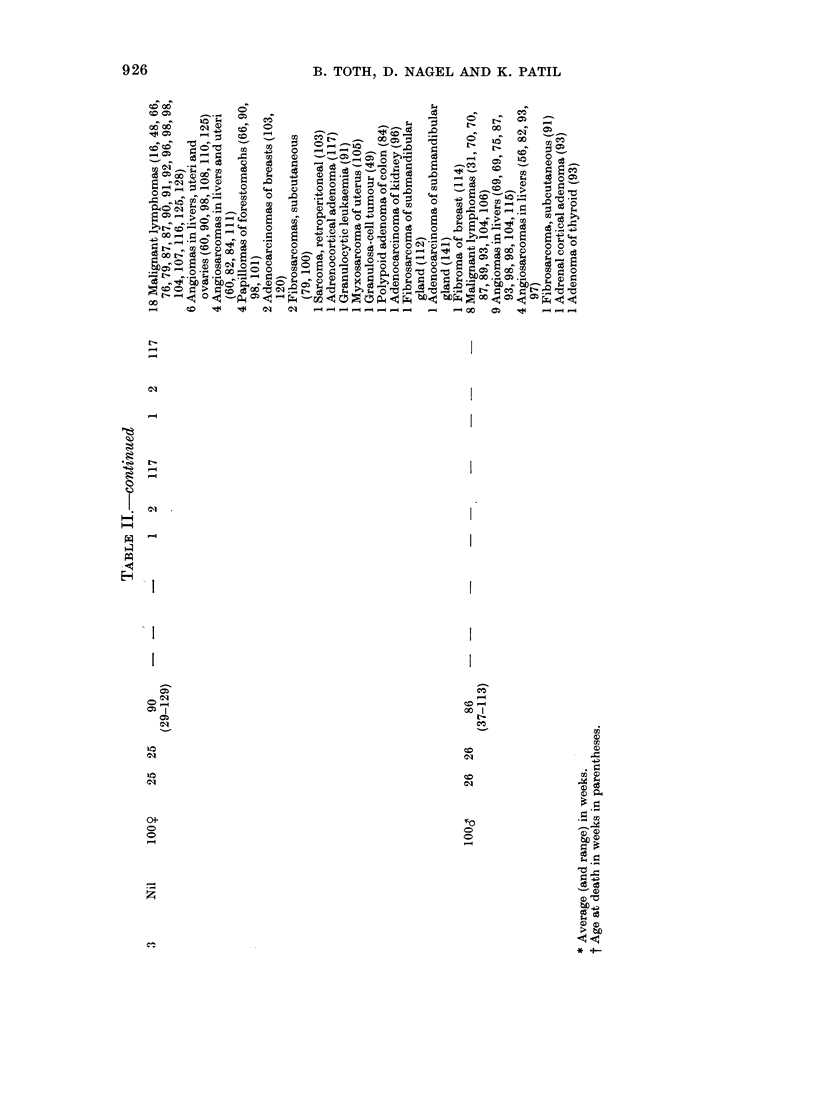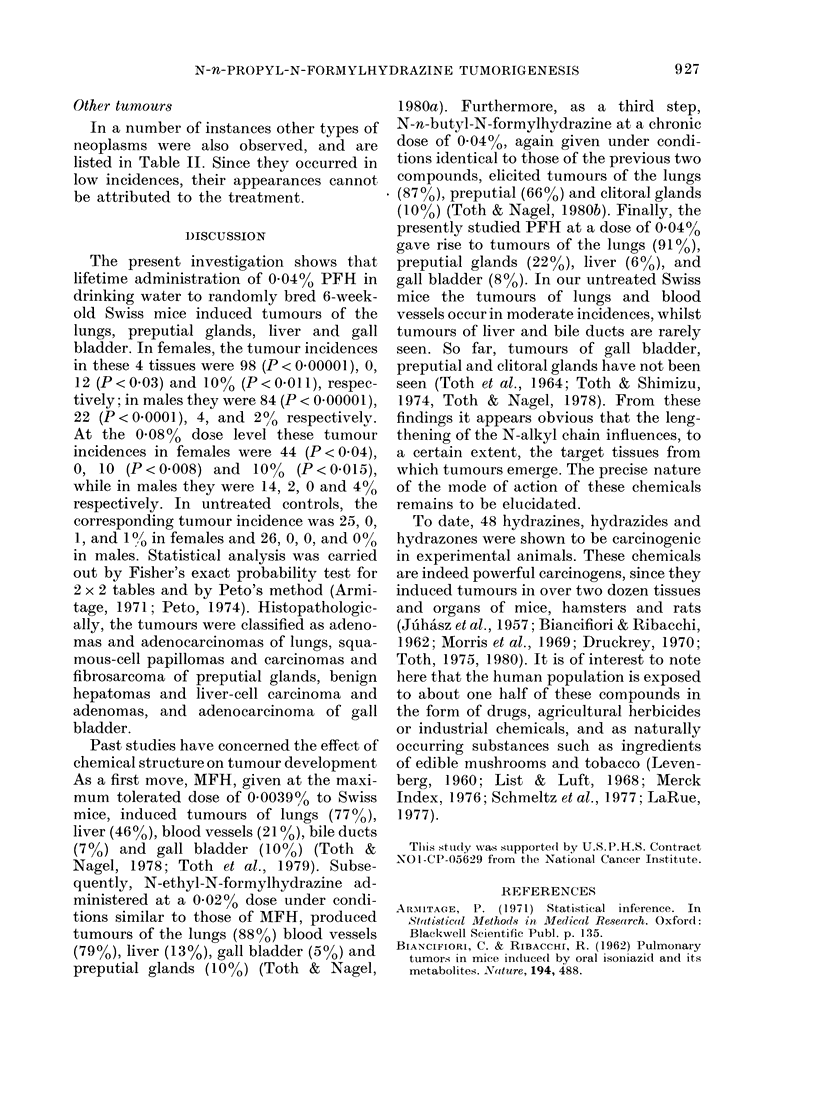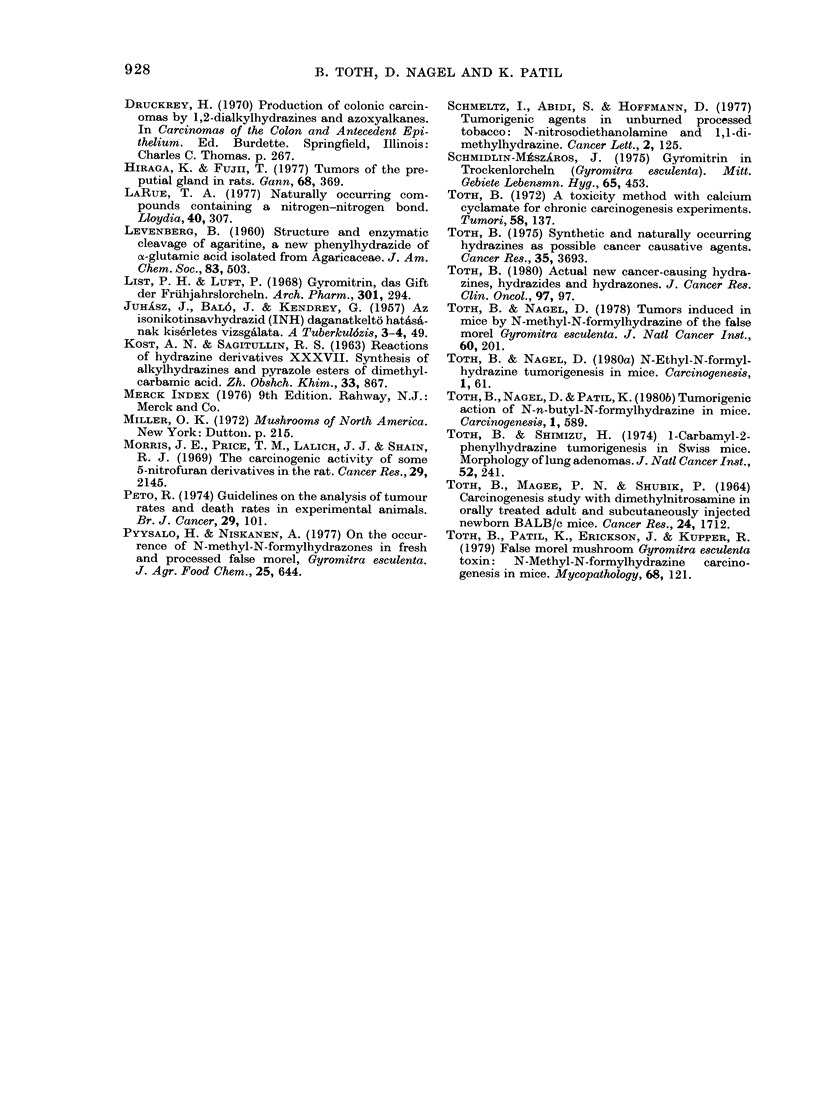# Tumorigenesis by N-n-propyl-N-formylhydrazine in mice.

**DOI:** 10.1038/bjc.1980.341

**Published:** 1980-12

**Authors:** B. Toth, D. Nagel, K. Patil

## Abstract

Continuous administration of 0.04% N-n-propyl-N-formylhydrazine (PFH) for life in drinking water to 6-week-old randomly bred Swiss mice induced tumours of the lungs, preputial glands, liver and gallbladder. The tumour incidences in these 4 tissues were 91, 22, 8 and 6%, whereas in the untreated controls they were 25, 0, 0.5 and 0.5%, respectively. The higher dose of 0.08% PFH, given under identical conditions, induced only tumours of the lungs, liver and gall bladder in low incidences, since the compound was too toxic for the mice. Histopathologically, the tumours were classified as adenomas and adenocarcinomas of the lungs, squamous-cell papillomas, and carcinomas and fibrosarcoma of preputial glands, benign hepatomas and liver-cell carcinoma, as well as adenomas and adenocarcinoma of the gall bladder. The investigation is part of our structure/activity relationship inquiry aimed at revealing the mechanism of action of the N-alkyl-N-formylhydrazine series of chemicals.


*Br. J. Cancer* (1980) **42**, 922

### TUMORIGENESIS BY N-*n*-PROPYL-N-FORMYLHYDRAZINE IN MICE

B. TOTH, D. NAGEL AND K. PATIL

*From The Eppley Institute for Research in Cancer, University of Nebraska Medical Centre, Omaha, Nebraska* 68105, *U.S.A.*

Received 28 July 1980 Accepted 17 September 1980

**Summary.—Continuous administration of 0·04% N-*n*-propyl-N-formylhydrazine (PFH) for life in drinking water to 6-week-old randomly bred Swiss mice induced tumours of the lungs, preputial glands, liver and gall bladder. The tumour incidences in these 4 tissues were 91, 22, 8 and 6%, whereas in the untreated controls they were 25, 0, 0·5 and 0·5%, respectively. The higher dose of 0·08% PFH, given under identical conditions, induced only tumours of the lungs, liver and gall bladder in lower incidences, since the compound was too toxic for the mice. Histopathologically, the tumours were classified as adenomas and adenocarcinomas of the lungs, squamous-cell papillomas, and carcinomas and fibrosarcoma of preputial glands, benign hepatomas and liver-cell carcinoma, as well as adenomas and adenocarcinoma of the gall bladder.**

**The investigation is part of our structure/activity relationship inquiry aimed at revealing the mechanism of action of the N-alkyl-N-formylhydrazine series of chemicals.**

THE PRESENT carcinogenesis study with N-*n*-propyl-N-formylhydrazine (PFH) in mice is a continuation of a structure/activity inquiry conducted with substituted hydrazines. Earlier investigations were performed in this species with structural homologues of PFH, including N-methyl-N-formylhydrazine (MFH), N-ethyl-N-formylhydrazine and N-*n*-butyl-N-formylhydrazine (Toth & Nagel, 1978; Toth *et al.*, 1979; Toth & Nagel, 1908*a*, *b*). At the beginning, we selected MFH as the first step in our inquiry because this chemical is an ingredient of the edible false morel mushroom *Gyromitra esculenta* (List & Luft, 1968; Schmidlin-Mészáros, 1975; Pyysalo & Niskanen, 1977). It is known that the human population is exposed to some extent, in certain areas, to this hazardous chemical (Miller, 1972). Subsequently, other N-alkyl-N-formylhydrazines were investigated to determine the possible relationship between chemical structure and tumour development at specific organ sites.

The current study proves the carcinogenicity of PFH given to Swiss mice at maximum tolerated dose levels in drinking water for life.

### MATERIALS AND METHODS

Swiss albino mice from the colony randomly bred by us since 1951 were used. They were housed in plastic cages with granular cellulose bedding, separated according to sex in groups of 5, and given Wayne-Lab blox diet in regular pellets (Allied Mills, Inc., Chicago, Illinois) and tap water or the chemical solution *ad libitum*.

The chemical used was N-*n*-propyl-N-formylhydrazine (PFH; Figure); mol. wt, 102·14; b.p., 98–100°C at 12 mm; purity >97%. PFH was synthesized in this laboratory in the following way (Kost & Sagitullin, 1963):

n-*Propylhydrazine*.—To a vigorously stirred solution of 1·0 kg (26·5 mol) of hydrazine monohydrate was added dropwise 285 g (2·3 mol) of *n*-propyl bromide. During the addition and 3 h after, the temperature was maintained at 45°C. The reaction mixture was con-

FIGURE—Chemical structure of N-*n*-propyl-N-formylhydrazine (PFH).

TABLE I.—*Treatment and survival rate in N-*n*-propyl-N-formylhydrazine (PFH)-treated and control Swiss mice*

| Group | PHF in drinking water for life | Initial No. and sex of mice | No. of survivors (age in weeks) | | | | | | | | | | | | | | |
|---|---|---|---|---|---|---|---|---|---|---|---|---|---|---|---|---|---|
| | | | 10 | 20 | 30 | 40 | 50 | 60 | 70 | 80 | 90 | 100 | 110 | 120 | 130 | 140 | 150 |
| 1 | 0·08% | 50♀ | 50 | 49 | 40 | 23 | 19 | 13 | 3 | — | | | | | | | |
| | | 50♂ | 50 | 37 | 11 | 2 | — | | | | | | | | | | |
| 2 | 0·04% | 50♀ | 50 | 50 | 50 | 50 | 45 | 35 | 18 | 2 | — | | | | | | |
| | | 50♂ | 50 | 48 | 45 | 32 | 23 | 5 | — | | | | | | | | |
| 3 | Nil | 100♀ | 100 | 99 | 98 | 92 | 83 | 77 | 70 | 55 | 45 | 30 | 16 | 6 | 1 | 1 | — |
| | | 100♂ | 100 | 100 | 100 | 96 | 91 | 81 | 63 | 44 | 28 | 11 | 4 | 1 | — | | |

that the treatments at both dose levels significantly shortened the survival time. The number and percentages of animals with tumours and their ages at death (latent periods) are summarized in Table II. The 4 statistically significant neoplasms were found in lungs, preputial glands, liver, and gall bladder, and are described in detail below:

*Lung tumours*

Of the females treated with the high dose, 22 (44%) developed 252 tumours in these organs. Of these, 19 mice had 188 adenomas and 3 mice had 50 adenomas and 14 adenocarcinomas. In the males treated with the high dose, 7 (14%) developed 54 adenomas.

Of the females treated with the low dose, 49 (98%) developed 751 tumours of the lungs. Of these, 18 mice had 177 adenomas and 31 mice developed 461 adenomas and 113 adenocarcinomas. In the males treated with the low dose, 42 (84%) developed 546 tumours in the lungs. Of these, 26 mice had 191 adenomas and 16 mice had 300 adenomas and 55 adenocarcinomas.

Macroscopically and histologically these lesions were similar to those described in this laboratory in this mouse strain (Toth & Shimizu, 1974; Toth *et al.*, 1979).

*Preputial-gland tumours*

Of the males treated with the high dose, 1 (2%) developed a squamous-cell carcinoma in this organ.

Of the males treated with the low dose, 11 (22%) developed tumours in this gland. Of these, 2 were classified as squamous-cell papillomas, 8 as squamous-cell carcinomas and the remaining one as a squamous-cell carcinoma jointly with a fibrosarcoma.

Grossly and histologically the above lesions were similar to those described earlier by other investigators (Hiraga & Fujii, 1977).

*Liver tumours*

Of the females treated with the high dose, 5 (10%) developed tumours in this organ. Of these, 4 were classified as benign hepatomas and the remaining one as a liver-cell carcinoma.

Of the mice treated with the low dose, 6 females (12%) and 2 males (4%) developed benign hepatomas.

Grossly and histopathologically the induced tumours were similar to those described earlier by us (Toth *et al.*, 1964).

*Gall-bladder tumours*

Of the females treated with the high dose, 5 (10%) developed tumours in this organ. Of these, 4 were classified as adenomas and the remaining one as an adenocarcinoma. In the males treated with the high dose, 2 (4%) developed adenomas of the gall bladder.

Of the mice treated with the low dose, 5 females (10%) and 1 male (2%) developed benign adenomas of this organ.

Macroscopically and histologically these tumours were similar to those published previously by us in other experiments (Toth & Nagel, 1978; Toth *et al.*, 1979).

### *Other tumours*

In a number of instances other types of neoplasms were also observed, and are listed in Table II. Since they occurred in low incidences, their appearances cannot be attributed to the treatment.

### DISCUSSION

The present investigation shows that lifetime administration of 0·04% PFH in drinking water to randomly bred 6-week-old Swiss mice induced tumours of the lungs, preputial glands, liver and gall bladder. In females, the tumour incidences in these 4 tissues were 98 ($P < 0{\cdot}00001$), 0, 12 ($P < 0{\cdot}03$) and 10% ($P < 0{\cdot}011$), respectively; in males they were 84 ($P < 0{\cdot}00001$), 22 ($P < 0{\cdot}0001$), 4, and 2% respectively. At the 0·08% dose level these tumour incidences in females were 44 ($P < 0{\cdot}04$), 0, 10 ($P < 0{\cdot}008$) and 10% ($P < 0{\cdot}015$), while in males they were 14, 2, 0 and 4% respectively. In untreated controls, the corresponding tumour incidence was 25, 0, 1, and 1% in females and 26, 0, 0, and 0% in males. Statistical analysis was carried out by Fisher's exact probability test for $2 \times 2$ tables and by Peto's method (Armitage, 1971; Peto, 1974). Histopathologically, the tumours were classified as adenomas and adenocarcinomas of lungs, squamous-cell papillomas and carcinomas and fibrosarcoma of preputial glands, benign hepatomas and liver-cell carcinoma and adenomas, and adenocarcinoma of gall bladder.

Past studies have concerned the effect of chemical structure on tumour development As a first move, MFH, given at the maximum tolerated dose of 0·0039% to Swiss mice, induced tumours of lungs (77%), liver (46%), blood vessels (21%), bile ducts (7%) and gall bladder (10%) (Toth & Nagel, 1978; Toth *et al.*, 1979). Subsequently, N-ethyl-N-formylhydrazine administered at a 0·02% dose under conditions similar to those of MFH, produced tumours of the lungs (88%) blood vessels (79%), liver (13%), gall bladder (5%) and preputial glands (10%) (Toth & Nagel, 1980*a*). Furthermore, as a third step, N-*n*-butyl-N-formylhydrazine at a chronic dose of 0·04%, again given under conditions identical to those of the previous two compounds, elicited tumours of the lungs (87%), preputial (66%) and clitoral glands (10%) (Toth & Nagel, 1980*b*). Finally, the presently studied PFH at a dose of 0·04% gave rise to tumours of the lungs (91%), preputial glands (22%), liver (6%), and gall bladder (8%). In our untreated Swiss mice the tumours of lungs and blood vessels occur in moderate incidences, whilst tumours of liver and bile ducts are rarely seen. So far, tumours of gall bladder, preputial and clitoral glands have not been seen (Toth *et al.*, 1964; Toth & Shimizu, 1974, Toth & Nagel, 1978). From these findings it appears obvious that the lengthening of the N-alkyl chain influences, to a certain extent, the target tissues from which tumours emerge. The precise nature of the mode of action of these chemicals remains to be elucidated.

To date, 48 hydrazines, hydrazides and hydrazones were shown to be carcinogenic in experimental animals. These chemicals are indeed powerful carcinogens, since they induced tumours in over two dozen tissues and organs of mice, hamsters and rats (Júhász *et al.*, 1957; Biancifiori & Ribacchi, 1962; Morris *et al.*, 1969; Druckrey, 1970; Toth, 1975, 1980). It is of interest to note here that the human population is exposed to about one half of these compounds in the form of drugs, agricultural herbicides or industrial chemicals, and as naturally occurring substances such as ingredients of edible mushrooms and tobacco (Levenberg, 1960; List & Luft, 1968; Merck Index, 1976; Schmeltz *et al.*, 1977; LaRue, 1977).

This study was supported by U.S.P.H.S. Contract NO1-CP-05629 from the National Cancer Institute.

### REFERENCES

ARMITAGE, P. (1971) Statistical inference. In *Statistical Methods in Medical Research.* Oxford: Blackwell Scientific Publ. p. 135.

BIANCIFIORI, C. & RIBACCHI, R. (1962) Pulmonary tumors in mice induced by oral isoniazid and its metabolites. *Nature*, **194**, 488.

DRUCKREY, H. (1970) Production of colonic carcinomas by 1,2-dialkylhydrazines and azoxyalkanes. In *Carcinomas of the Colon and Antecedent Epithelium.* Ed. Burdette. Springfield, Illinois: Charles C. Thomas. p. 267.

HIRAGA, K. & FUJII, T. (1977) Tumors of the preputial gland in rats. *Gann*, **68**, 369.

LARUE, T. A. (1977) Naturally occurring compounds containing a nitrogen–nitrogen bond. *Lloydia*, **40**, 307.

LEVENBERG, B. (1960) Structure and enzymatic cleavage of agaritine, a new phenylhydrazide of α-glutamic acid isolated from Agaricaceae. *J. Am. Chem. Soc.*, **83**, 503.

LIST, P. H. & LUFT, P. (1968) Gyromitrin, das Gift der Frühjahrslorcheln. *Arch. Pharm.*, **301**, 294.

JUHÁSZ, J., BALÓ, J. & KENDREY, G. (1957) Az isonikotinsavhydrazid (INH) daganatkeltö hatásának kisérletes vizsgálata. *A Tuberkulózis*, **3–4**, 49.

KOST, A. N. & SAGITULLIN, R. S. (1963) Reactions of hydrazine derivatives XXXVII. Synthesis of alkylhydrazines and pyrazole esters of dimethylcarbamic acid. *Zh. Obshch. Khim.*, **33**, 867.

MERCK INDEX (1976) 9th Edition. Rahway, N.J.: Merck and Co.

MILLER, O. K. (1972) *Mushrooms of North America.* New York: Dutton. p. 215.

MORRIS, J. E., PRICE, T. M., LALICH, J. J. & SHAIN, R. J. (1969) The carcinogenic activity of some 5-nitrofuran derivatives in the rat. *Cancer Res.*, **29**, 2145.

PETO, R. (1974) Guidelines on the analysis of tumour rates and death rates in experimental animals. *Br. J. Cancer*, **29**, 101.

PYYSALO, H. & NISKANEN, A. (1977) On the occurrence of N-methyl-N-formylhydrazones in fresh and processed false morel, *Gyromitra esculenta.* *J. Agr. Food Chem.*, **25**, 644.

SCHMELTZ, I., ABIDI, S. & HOFFMANN, D. (1977) Tumorigenic agents in unburned processed tobacco: N-nitrosodiethanolamine and 1,1-dimethylhydrazine. *Cancer Lett.*, **2**, 125.

SCHMIDLIN-MÉSZÁROS, J. (1975) Gyromitrin in Trockenlorcheln (*Gyromitra esculenta*). *Mitt. Gebiete Lebensmn. Hyg.*, **65**, 453.

TOTH, B. (1972) A toxicity method with calcium cyclamate for chronic carcinogenesis experiments. *Tumori*, **58**, 137.

TOTH, B. (1975) Synthetic and naturally occurring hydrazines as possible cancer causative agents. *Cancer Res.*, **35**, 3693.

TOTH, B. (1980) Actual new cancer-causing hydrazines, hydrazides and hydrazones. *J. Cancer Res. Clin. Oncol.*, **97**, 97.

TOTH, B. & NAGEL, D. (1978) Tumors induced in mice by N-methyl-N-formylhydrazine of the false morel *Gyromitra esculenta. J. Natl Cancer Inst.*, **60**, 201.

TOTH, B. & NAGEL, D. (1980*a*) N-Ethyl-N-formylhydrazine tumorigenesis in mice. *Carcinogenesis*, **1**, 61.

TOTH, B., NAGEL, D. & PATIL, K. (1980*b*) Tumorigenic action of N-*n*-butyl-N-formylhydrazine in mice. *Carcinogenesis*, **1**, 589.

TOTH, B. & SHIMIZU, H. (1974) 1-Carbamyl-2-phenylhydrazine tumorigenesis in Swiss mice. Morphology of lung adenomas. *J. Natl Cancer Inst.*, **52**, 241.

TOTH, B., MAGEE, P. N. & SHUBIK, P. (1964) Carcinogenesis study with dimethylnitrosamine in orally treated adult and subcutaneously injected newborn BALB/c mice. *Cancer Res.*, **24**, 1712.

TOTH, B., PATIL, K., ERICKSON, J. & KUPPER, R. (1979) False morel mushroom *Gyromitra esculenta* toxin: N-Methyl-N-formylhydrazine carcinogenesis in mice. *Mycopathology*, **68**, 121.